# Formulation, Characterization, and *in Vivo* Evaluation of Celecoxib-PVP Solid Dispersion Nanoparticles Using Supercritical Antisolvent Process

**DOI:** 10.3390/molecules191220325

**Published:** 2014-12-04

**Authors:** Eun-Sol Ha, Gwang-Ho Choo, In-Hwan Baek, Min-Soo Kim

**Affiliations:** 1 College of Pharmacy, Pusan National University, Busan 609-735, Korea; E-Mails: edel@pusan.ac.kr (E.-S.H.); jimasd@pusan.ac.kr (G.-H.C.); 2 College of Pharmacy, Kyungsung University, Busan 608-736, Korea; E-Mail: baek@ks.ac.kr

**Keywords:** solid dispersion, bioavailability, celecoxib, nanoparticles, supercritical antisolvent

## Abstract

The aim of this study was to develop celecoxib-polyvinylpyrrolidone (PVP) solid dispersion nanoparticles with and without surfactant using the supercritical antisolvent (SAS) process. The effect of different surfactants such as gelucire 44/14, poloxamer 188, poloxamer 407, Ryoto sugar ester L1695, and d-α-tocopheryl polyethylene glycol 1000 succinate (TPGS) on nanoparticle formation and dissolution as well as oral absorption of celecoxib-PVP K30 solid dispersion nanoparticles was investigated. Spherical celecoxib solid dispersion nanoparticles less than 300 nm in size were successfully developed using the SAS process. Analysis by differential scanning calorimetry and powder X-ray diffraction showed that celecoxib existed in the amorphous form within the solid dispersion nanoparticles fabricated using the SAS process. The celecoxib-PVP-TPGS solid dispersion nanoparticles significantly enhanced *in vitro* dissolution and oral absorption of celecoxib relative to that of the unprocessed form. The area under the concentration-time curve (AUC_0→24 h_) and peak plasma concentration (C_max_) increased 4.6 and 5.7 times, respectively, with the celecoxib-PVP-TPGS formulation. In addition, *in vitro* dissolution efficiency was well correlated with *in vivo* pharmacokinetic parameters. The present study demonstrated that formulation of celecoxib-PVP-TPGS solid dispersion nanoparticles using the SAS process is a highly effective strategy for enhancing the bioavailability of poorly water-soluble celecoxib.

## 1. Introduction

Celecoxib, 4-[5-(4-methylphenyl)-3-(trifluoromethyl)pyrazol-1-yl]benzenesulfonamide, is a poorly water-soluble drug belonging to the class of selective cyclooxygenase-2 (COX-2) inhibitors and is clinically used in the treatment of acute pain, rheumatoid arthritis, and osteoarthritis [1]. Celecoxib with an acid dissociation constant (pK_a_) of 11.1 belongs to the biopharmaceutics classification system (BSC) class II drug category because of its low solubility and high permeability [2]. Various formulations of celecoxib such as self-microemulsifying drug delivery systems (SMEDDS), microparticles, nanoparticles, silica-lipid hybrid microcapsules, and surface solid dispersions have been studied to evaluate their potential to enhance dissolution of celecoxib [3,4,5,6,7,8].

Solid dispersion is well established as a formulation system for enhancing the bioavailability of poorly water-soluble active pharmaceutical ingredients (APIs). Generally, solid dispersions consist of an API and a hydrophilic polymer such as hydroxypropyl methylcellulose (HPMC), polyvinylpyrrolidone (PVP), or polyethylene glycol (PEG) [9,10,11]. Most poorly water-soluble APIs exist in an amorphous form within the solid dispersion, thereby enhancing their dissolution and oral absorption by attaining a highly supersaturated state above their equilibrium solubility. Furthermore, ternary solid dispersions consisting of API, polymer, and surfactant can further enhance dissolution and *in vivo* performance of APIs compared to binary solid dispersions [12]. Our group recently reported that of the 71 combination formulations we evaluated, the most efficient ternary solid dispersion for enhanced bioavailability of sirolimus was the HPMC/d- α-tocopheryl polyethylene glycol 1000 succinate (TPGS) followed by the HPMC/Sucroester 15 [13]. Solid dispersions containing polymer and/or surfactant can be manufactured on the principle of solvent evaporation, melting, and/or solvent-mediated melting. It has been reported that solid dispersion nanoparticles can be manufactured using supercritical fluid technology. Supercritical carbon dioxide (Pc = 7.38 MPa, Tc = 31.1 °C) is widely used as a solvent or antisolvent in the field of nanoparticle formation because it is non-toxic and non-flammable. Supercritical carbon dioxide can therefore be considered a green solvent alternative to potential toxic organic solvents in the pharmaceutical industry. The nanoparticle manufacturing process using supercritical carbon dioxide was developed based on the role of the supercritical carbon dioxide in the process as either a solvent (rapid expansion of supercritical solutions) or an antisolvent (supercritical antisolvent process; SAS). Yasuji *et al.* reviewed particle design of poorly water-soluble APIs using supercritical fluid technologies [14]. Solid dispersion nanoparticles manufactured with hydrophilic polymers and surfactants using the SAS process significantly improved the solubility, dissolution, and oral bioavailability of poorly water-soluble APIs such as atorvastatin calcium, azithromycin, dutasteride, lercanidipine, nilotinib, valsartan, tadalafil and telmisartan [15,16,17,18,19,20,21,22,23].

The aim of this study was to develop celecoxib-PVP solid dispersion nanoparticles with and without surfactant using the SAS process and to evaluate their potential to enhance the dissolution and oral bioavailability of celecoxib. Recently, Abu-Diak *et al.* reported that the recrystallization of celecoxib from supersaturated solution was significantly inhibited by PVP in a concentration-dependent manner [24]. Therefore, we used PVP K30 as a hydrophilic polymer in this study. The effect of different surfactants including gelucire 44/14, poloxamer 188, poloxamer 407, Ryoto sugar ester L1695, and TPGS on the nanoparticle formation, dissolution, and oral absorption of celecoxib-PVP K30 solid dispersion nanoparticles was investigated. An *in vitro*-in vivo correlation (IVIVC) study was also conducted using the *in vitro* dissolution data and *in vivo* pharmacokinetic parameters.

## 2. Results and Discussion

**Figure 1:**
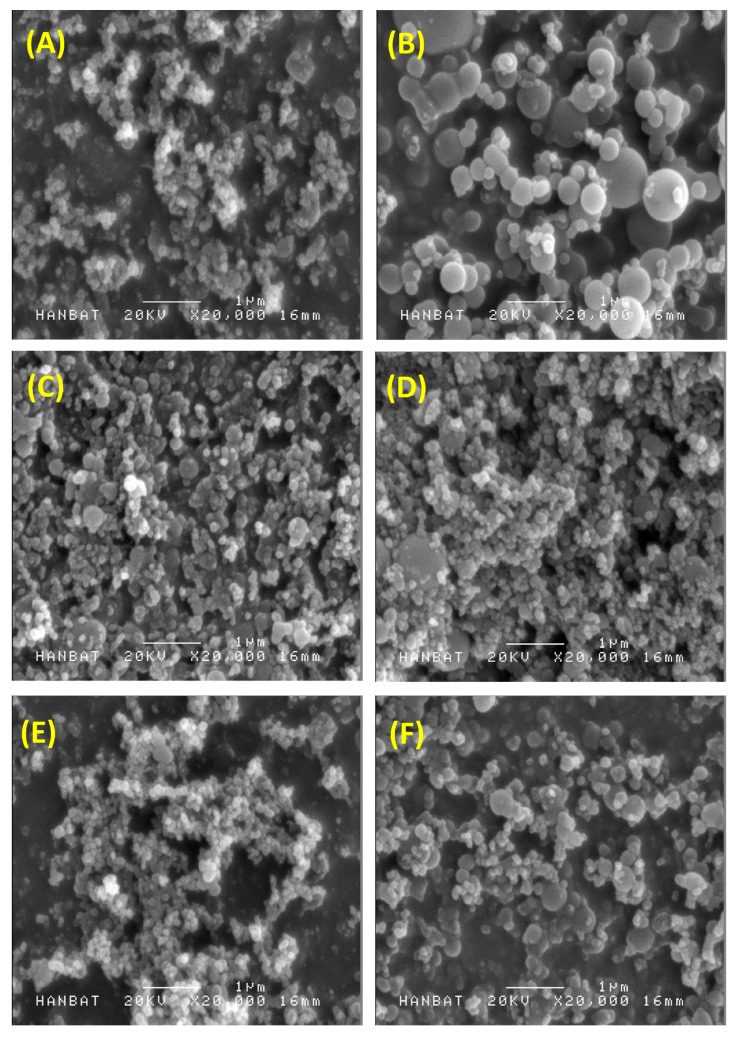
Scanning electron micrographs of celecoxib-polyvinylpyrrolidone (celecoxib-PVP) K30 solid dispersion nanoparticles prepared using the supercritical antisolvent (SAS) process. (**A**) celecoxib-PVP K30 (2:8); (**B**) celecoxib-PVP K30-gelucire 44/14; (**C**) celecoxib-PVP K30-poloxamer 188; (**D**) celecoxib-PVP K30-poloxamer 407; (**E**) celecoxib-PVP K30-Ryoto sugar ester L1695; and (**F**) celecoxib-PVP K30-d-α-tocopheryl polyethylene glycol 1000 succinate (K30-TPGS).

**Figure 2:**
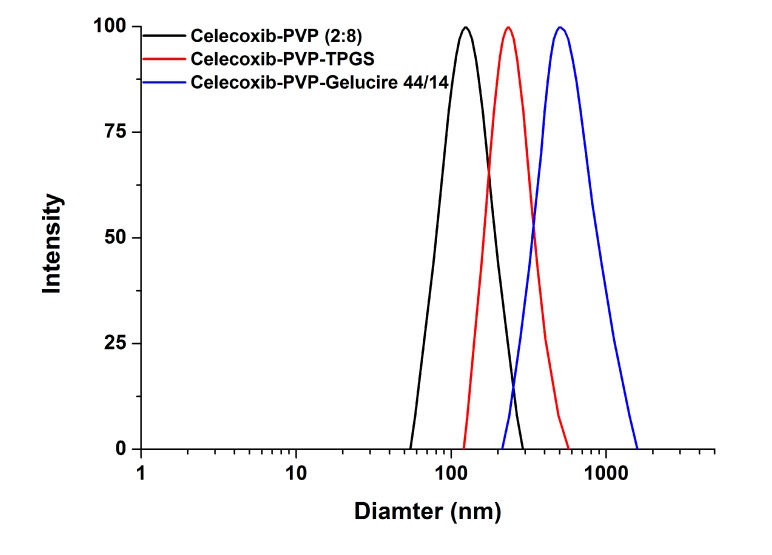
The particle size distribution of celecoxib-PVP K30 solid dispersion nanoparticles prepared using the SAS process.

In this study, celecoxib-PVP K30 solid dispersion nanoparticles were formulated using the SAS process with the aim of enhancing the dissolution and oral absorption of celecoxib. Figure 1 shows the SEM images of the solid dispersion nanoparticles and Table 1 summarizes the mean particle size and specific surface area of solid dispersion nanoparticles. All celecoxib-PVP K30 solid dispersion nanoparticles had regular spherical shape with particle size range of 150–158 nm and specific surface area of 78–81 m^2^/g, indicating there was no significant difference between the celecoxib-PVP K30 solid dispersion nanoparticle formulations. The ratio of celecoxib/PVP K30 also did not appear to influence the morphology and particle size of solid dispersion nanoparticles prepared by the SAS process. However, the mean particle size and specific surface area of solid dispersion nanoparticles were significantly affected by addition of the surfactant (Figure 2).

In particular, the particles of the celecoxib-PVP K30-gelucire 44/14 solid dispersion nanoparticles had a regular spherical shape with mean particle size of 610.3 nm and specific surface area of 21.2 m^2^/g. As shown in Table 1, the mean particle size and specific surface area of solid dispersion nanoparticles increased and decreased, respectively, following addition of the surfactant. The increased mean particle size and aggregation of solid dispersion nanoparticles might be due to the fusion of the surfactant, resulting in lower melting temperature. The melting points of all surfactants tested were below 56 °C as stated in the material and methods section. Similar results were previously reported [15,16,17,18]. Nevertheless, solid dispersion nanoparticles with surfactants (with the exception of gelucire 44/14) showed mean particle sizes below 300 nm.

**Figure 3:**
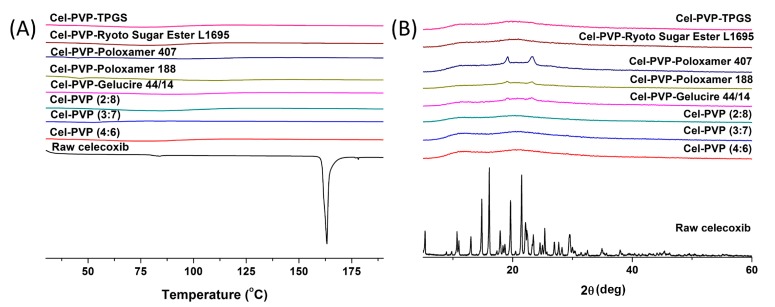
Differential scanning calorimetry thermograms (**A**) and powder X-ray diffraction patterns (**B**) of celecoxib-PVP K30 solid dispersion nanoparticles prepared using the SAS process. Cel: celecoxib.

The crystal state of celecoxib within the solid dispersion nanoparticles was determined by DSC curves and PXRD patterns and is shown in Figure 3. Raw celecoxib showed a sharp endothermic peak at about 163 °C. In addition, characteristic diffraction patterns indicating crystalline celecoxib were observed at 2θ values of 5.31°, 10.68°, 13.00°, 14.83°, 16.08°, 19.63°, 21.49°, 22.13°, 25.36°, and 29.48°. However, the melting peak and diffraction patterns of crystalline celecoxib were not observed for all solid dispersion nanoparticle formulations. In fact, celecoxib exists in the amorphous form in solid dispersion nanoparticle fabricated by the SAS process.

**Table 1:** Formulation, particle size, and specific surface area of celecoxib-polyvinylpyrrolidone (celecoxib-PVP) solid dispersion nanoparticles prepared using the supercritical antisolvent (SAS) process.

Formulation (Weight)	Drug Content (%) ^#^	Mean Particle Size (nm)	Specific Surface Area (m^2^/g)
Celecoxib:PVP K30 = 4:6	90.6 ± 2.3	158.3 ± 19.8	78.1 ± 1.3
Celecoxib:PVP K30 = 3:7	92.7 ± 3.1	150.1 ± 15.5	80.4 ± 1.4
Celecoxib:PVP K30 = 2:8	94.5 ± 1.9	155.3 ± 12.1	81.2 ± 1.8
Celecoxib:PVP K30:Gelucire 44/14 = 2:5:3	96.9 ± 1.1	610.3 ± 71.2	21.2 ± 1.2
Celecoxib:PVP K30:Poloxamer 188 = 2:5:3	95.3 ± 1.0	275.1 ± 33.4	40.1 ± 1.1
Celecoxib:PVP K30:Poloxamer 407 = 2:5:3	94.5 ± 1.3	282.7 ± 40.5	36.8 ± 1.1
Celecoxib:PVP K30:Ryoto sugar ester L1695 = 2:5:3	95.8 ± 2.4	220.1 ± 23.4	49.7 ± 1.3
Celecoxib:PVP K30:TPGS = 2:5:3	95.1 ± 1.8	290.8 ± 44.7	29.2 ± 1.0

^#^ The celecoxib concentration within the solid dispersion nanoparticles was determined by dissolving about 30 mg of solid dispersion nanoparticles in 100 mL ethanol, filtering aliquots using a 0.45-μm syringe filter, and analyzing concentration with an HPLC system The drug content (%) = weight of the drug in nanoparticles/weight of the feeding excipients and drug × 100. Data are expressed as the mean ± standard deviation (*n* = 3).

**Figure 4:**
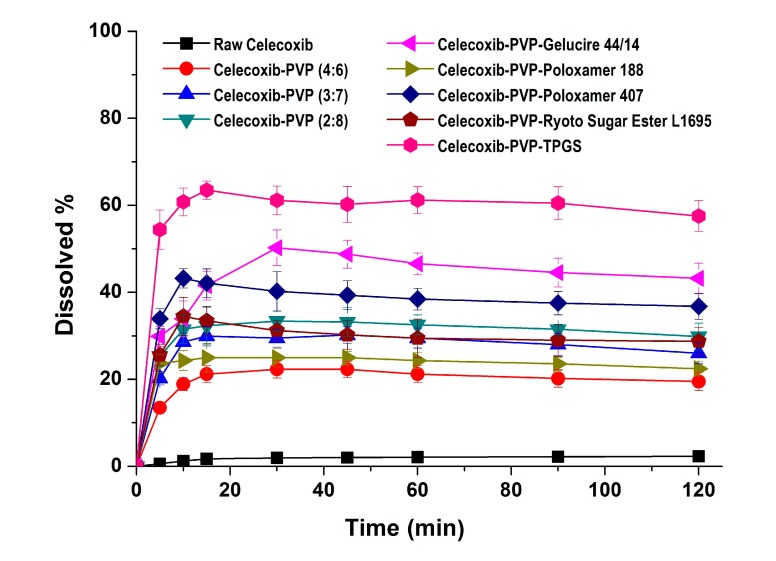
Dissolution profiles of celecoxib-PVP K30 solid dispersion nanoparticles prepared using the SAS process in pH 1.2 dissolution medium. Data are expressed as the mean ± standard deviation (*n* = 3).

**Figure 5:**
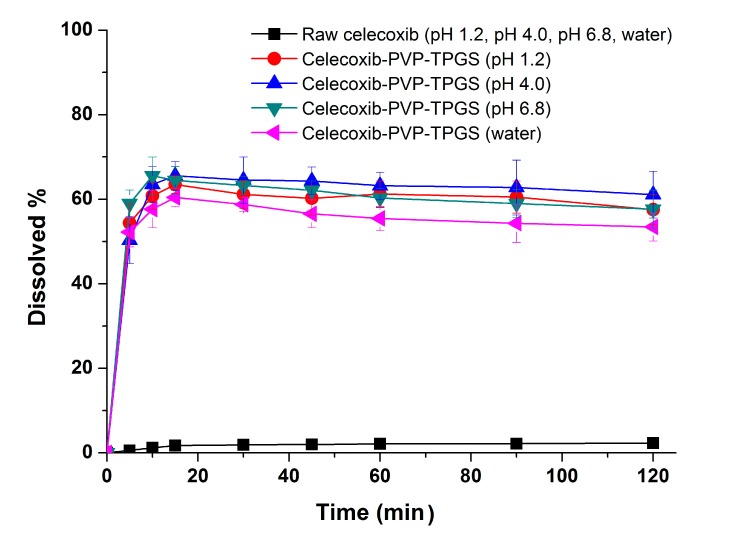
Effect of dissolution media on the dissolution of celecoxib-PVP K30-TPGS solid dispersion nanoparticles prepared using the SAS process. Data are expressed as the mean ± standard deviation (*n* = 3).

In the dissolution studies (Figure 4), raw celecoxib showed very low dissolution because of its poor water solubility. However, the dissolution of celecoxib was significantly increased by solid dispersion nanoparticles. The dissolution of drug from celecoxib-PVP (2:8) solid dispersion nanoparticles had a maximum rate of 33.0% within 0.25 h, and gradually decreased to 26.3% after 2 h (Figure 4). The maximum dissolution and dissolution percentage of celecoxib at 2 h, increased with increasing PVP ratio within nanoparticles, but there were no significant differences between PVP solid dispersion nanoparticles (4:6, 3:7, and 2:8). The enhanced dissolution properties of celecoxib might be due to the formation of amorphous celecoxib in PVP solid dispersion nanoparticles prepared by the SAS process and the specific interaction of PVP and celecoxib in forming hydrogen bonds [25]. It has also been previously reported that recrystallization of celecoxib from supersaturated solution was significantly inhibited by PVP in a concentration-dependent manner [24,25]. Gupta *et al.* reported that the solubility of amorphous celecoxib was 20.43 μg/mL in PVP aqueous solution and 4.57 μg/mL in water, indicating that PVP enhances solubilization and stabilization of amorphous celecoxib [26]. The dissolution of celecoxib was also enhanced by incorporated of surfactant within the solid dispersion nanoparticles. In particular, the maximum celecoxib dissolution in celecoxib-PVP K30-TPGS solid dispersion nanoparticles was 64%, and dissolution at 2 h was 56%. The dissolution percentage of celecoxib at 2 h ranked by the SNK test ranged as follows: celecoxib-PVP K30-poloxamer 188 < celecoxib-PVP K30-Ryoto sugar ester L1695 < celecoxib-PVP K30-poloxamer 407 < celecoxib-PVP K30-gelucire 44/14 < celecoxib-PVP K30-TPGS solid dispersion nanoparticle. In solubility studies, the most effective surfactant tested was TPGS, followed by gelucire 44/14 and poloxamer 407, as shown in Table 2. Dissolution of celecoxib from solid dispersion nanoparticles with surfactant at 2 h was in fact related to the solubilization capability of the surfactant used. As reported in our previous study, the solubility of celecoxib was significantly increased by TPGS via micellar solubilization at a critical micelle concentration of 0.1 mg/mL [6]. Furthermore, it was reported that the recrystallization of celecoxib from amorphous state can be effectively inhibited by the combination of PVP and TPGS [27]. Therefore, the significant enhancement in both the dissolution rate and extent of dissolution of celecoxib can probably be attributed to the increased solubility induced by the PVP-TPGS stabilized amorphous form, the enhanced solubility by micellar solubilization of TPGS, and/or the improved wettability of particle surface by PVP and surfactant. In addition, celecoxib-PVP K30-TPGS solid dispersion nanoparticles showed higher dissolution above 60% at different dissolution media pH (1.2, 4.0, 6.8 and water), as shown in Figure 5. Interestingly, the dissolution of celecoxib from celecoxib-PVP K30-TPGS solid dispersion nanoparticles was not influenced by the pH of dissolution media. The solubility of celecoxib was not influenced by the physiological pH condition (1.0–7.5) since the pK_a_ of celecoxib is 11.1. In fact, raw celecoxib showed very low dissolution of below 3% in different pH dissolution media (pH 1.2, 4.0, 6.8 and water). To determine the IVIVC for celecoxib, the dissolution efficiency (DE%) as defined by Khan and Rhodes, was calculated using the dissolution profiles of celecoxib.

**Table 2:** Solubility of celecoxib in various surfactant solutions.

Surfactant	Solubility (μg/mL) ^#^
Raw celecoxib	3.9 ± 0.2
Gelucire 44/14	3100.1 ± 65.8
Poloxamer 188	14.8 ± 3.2
Poloxamer 407	2060.7 ± 40.3
Ryoto Sugar Ester L1695	1380.9 ± 54.2
TPGS	6522.3 ± 25.0

^#^ The solubility of celecoxib was measured in 1% surfactant solution at 37 °C. Data are expressed as the mean ± standard deviation (*n* = 3).

The dissolution efficiency for celecoxib-PVP solid dispersion nanoparticles was calculated from the area under the dissolution curves at 120 min and expressed as a percentage of the area of the rectangle resulting from 100% dissolution within the same period [28]. As shown in Table 3, celecoxib-PVP K30-TPGS solid dispersion nanoparticles showed the highest DE%.

**Table 3:** Dissolution efficiency of celecoxib-PVP solid dispersion nanoparticles prepared using the SAS process.

Formulation	DE (%) ^#^
Raw celecoxib	1.9 ± 0.2
Celecoxib:PVP K30 = 2:8	31.1 ± 2.6
Celecoxib:PVP K30: Gelucire 44/14 = 2:5:3	43.8 ± 3.0
Celecoxib:PVP K30:Poloxamer 188 = 2:5:3	23.6 ± 2.0
Celecoxib:PVP K30:Poloxamer 407 = 2:5:3	37.9 ± 2.8
Celecoxib:PVP K30:Ryoto Sugar Ester L1695 = 2:5:3	29.3 ± 3.1
Celecoxib:PVP K30:TPGS = 2:5:3	59.1 ± 3.3

^#^ Data are expressed as the mean ± standard deviation (*n* = 3).

**Figure 6:**
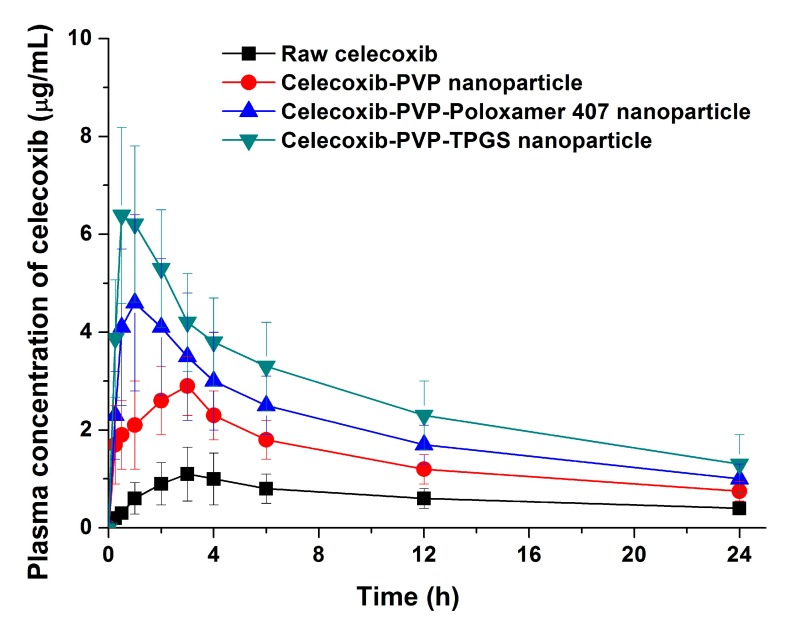
Plasma concentration-time profile of celecoxib in rats after oral administration of the raw celecoxib and celecoxib-PVP K30 solid dispersion nanoparticles prepared using the SAS process. Data are expressed as the mean ± standard deviation (*n* = 5).

**Figure 7:**
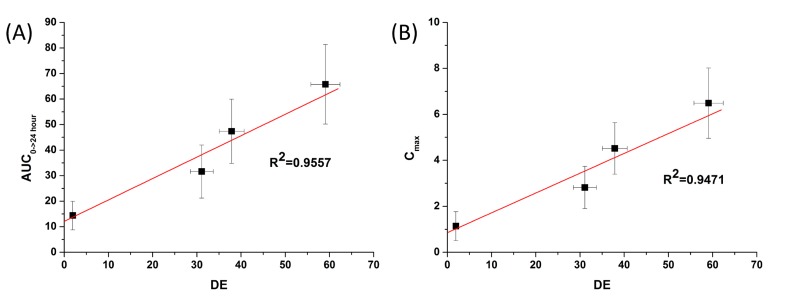
Correlation between the *in vitro* dissolution efficiency and *in vivo* pharmacokinetic parameters of celecoxib. (**A**) Area under the curve (AUC) and (**B**) C_max_.

The bioavailability of celecoxib solid dispersion nanoparticles and raw celecoxib was determined in SD rats. As shown in the plasma concentration–time curves (Figure 6), the C_max_ of celecoxib from solid dispersion nanoparticles was significantly increased compared to raw celecoxib. From the pharmacokinetic data, C_max_ and AUC_0→24 h_ of raw celecoxib was 1.14 μg/mL and 14.42 μg·h/mL, respectively (Table 4). As expected from the dissolution data, celecoxib-PVP K30-TPGS solid dispersion nanoparticles showed highest C_max_ and AUC_0→24 h_ values and were 4.6 and 5.7 times higher, respectively, that shown by raw celecoxib. Furthermore, ANOVA showed that there were significant differences among the samples (*p* < 0.05), which in order of increasing the C_max_ of celecoxib, were ranked by the SNK test as follows: raw celecoxib < celecoxib-PVP K30 (2:8) < celecoxib-PVP K30-poloxamer 407 < celecoxib-PVP K30-TPGS solid dispersion nanoparticle. Further studies of correlation between the *in vitro* dissolution data and *in vivo* pharmacokinetic parameters (Figure 7) showed *in vitro* DE was well correlated to *in vivo* C_max_, and AUC_0→24 h_ (R^2^ > 0.90). In fact, *in vitro* dissolution of celecoxib was increased by solid dispersion nanoparticles, resulting in increased oral bioavailability. This also implies that the oral bioavailability of celecoxib can be controlled by the *in vitro* dissolution property. This correlation was similar to previously reported IVIVC studies for celecoxib using SMEDDS formulation and silica-lipid hybrid formulation [4,5]. As reported in previous studies, IVIVC study using DE is an effective method for analyzing celecoxib in solid dispersion nanoparticles [18].

**Table 4:** Pharmacokinetic parameters of celecoxib-PVP solid dispersion nanoparticles prepared using the SAS process.

Formulation	AUC_0→24 h_ (μg·h/mL)	C_max_ (μg/mL)	T_max_ (h)
Raw celecoxib	14.42 ± 5.59	1.14 ± 0.63	4.0 ± 1.2
Celecoxib:PVP K30 = 2:8	31.63 ± 10.38 ^a^	2.82 ± 0.92 ^a^	3.6 ± 1.5
Celecoxib:PVP K30:Poloxamer 407 = 2:5:3	47.39 ± 12.56 ^ab^	4.52.6 ± 1.12 ^ab^	2.0 ± 1.0
Celecoxib:PVP K30:TPGS = 2:5:3	65.78 ± 15.56 ^a–c^	6.49 ± 1.53 ^a–c^	1.4 ± 1.1

^a^ indicates *p* < 0.05 *vs*. raw celecoxib; ^b^ indicates *p* < 0.05 *vs*. Celecoxib:PVP K30; ^c^ indicates *p* < 0.05 *vs*. Celecoxib:PVP K30:Poloxamer 407. Data are expressed as the mean ± standard deviation (*n* = 5).

Generally, supersaturatable formulations such as solid dispersions, raise the highly supersaturated state of poorly water-soluble APIs above their equilibrium solubility in *in vitro* dissolution medium and *in vivo* in the gastrointestinal tract [29]. To enhance the bioavailability of poorly water-soluble APIs by a supersaturatable system, the formulation must have the essential properties of generation and maintenance of the thermodynamically metastable supersaturated state [30]. Therefore, the precipitation of APIs must be inhibited by using a hydrophilic polymer and surfactant. In this study, the enhanced solubility of celecoxib induced by celecoxib-PVP-TPGS solid dispersion nanoparticles resulted in enhanced oral absorption through the gastrointestinal epithelial membrane. This formulation can also be applied to potentially enhance the bioavailability of other poorly water-soluble APIs.

## 3. Experimental Section

### 3.1. Materials

Celecoxib was obtained from Dong-A ST (Yongin, Korea). Gelucire 44/14 (melting point, 44 °C, Gattefossè, Saint-Priest, France), polyvinylpyrrolidone (PVP K30, BASF Co. Ltd., Ludwigshafen, Germany), poloxamer 188 (melting point: 52 °C, BASF Co. Ltd., Ludwigshafen, Germany), poloxamer 407 (melting point: 56 °C BASF Co. Ltd., Ludwigshafen, Germany), Ryoto sugar ester L1695 (melting point: 35–50 °C, Mitsubishi-Kagaku Foods Co., Tokyo, Japan), and d-α-tocopheryl polyethylene glycol 1000 succinate (melting point: 37 °C, TPGS, Eastman Co., Kingsport, TN, USA) were used. Hydrochlorothiazide was purchased from Sigma-Aldrich Co. Ltd (St. Louis, MO, USA). Acetonitrile, ethanol, and methanol were of high-performance liquid chromatography (HPLC) grade.

### 3.2. Preparation of Celecoxib-PVP K30 Solid Dispersion Nanoparticles

Celecoxib solid dispersion nanoparticles were manufactured by the SAS process using Thar SAS200 equipment (Thar Technologies, Pittsburgh, PA, USA) [31,32]. To study the effect of the celecoxib/PVP K30 ratio, celecoxib-PVP K30 nanoparticles were fabricated with 20%, 30%, and 40% drug loading concentrations. The drug solution was first prepared by dissolving celecoxib and PVP K30 in methanol at 5% solute concentration. Once the particle precipitation vessel reached steady state (above critical temperature and pressure), the drug solution was introduced into the particle precipitation vessel by an HPLC liquid pump. The SAS process was then performed under the following conditions, based on preliminary experiments [15]: temperature of precipitation vessel, 40 °C; pressure of precipitation vessel, 15 MPa; flow rate of drug solution, 1 mL/min; and flow rate of supercritical carbon dioxide, 11 g/min. To completely extract residual methanol, supercritical carbon dioxide was introduced into the precipitation vessel for 1 h, and the celecoxib solid dispersion nanoparticles were obtained from the precipitation vessel after depressurization to atmospheric pressure level. To investigate the effect of various surfactants on the dissolution and bioavailability of celecoxib, ternary solid dispersion nanoparticles of celecoxib-PVP K30 and surfactants were also prepared. Drug solutions were prepared by dissolving celecoxib, PVP K30, and the respective surfactant (with the exception of gelucire 44/14) in methanol at 5% solute concentration. For gelucire 44/14, the drug solution was prepared using a 1:1 mixture of methanol and dichloromethane. Gelucire 44/14, poloxamer 188, poloxamer 407, Ryoto sugar ester L1695, and TPGS were tested as surfactants. The nanoparticles were manufactured under the same conditions as described above for the formulation without surfactant. The formulation characteristics of celecoxib solid dispersion nanoparticles are presented in Table 1.

### 3.3. Characterization of Celecoxib-PVP K30 Solid Dispersion Nanoparticles

The morphology of celecoxib-PVP K30 solid dispersion nanoparticles was examined using a scanning electron microscope (SEM, JSM-6300, Jeol Ltd., Tokyo, Japan). After suspension of the nanoparticles in mineral oil by sonication for 20 min, the particle size of celecoxib-PVP K30 solid dispersion nanoparticles was determined by the dynamic light scattering method using a laser particle analyzer (BI-9000; Brookhaven, NY, USA). The specific surface area (m^2^/g) of celecoxib-PVP solid dispersion nanoparticles was measured by the Brunauer, Emmett, and Teller (BET) method with nitrogen as the adsorption gas using an Autosorb-1 instrument (Quantachrome GmbH, Odelzhausen, Germany). The crystalline state of celecoxib within the solid dispersion nanoparticle was characterized using a differential scanning calorimeter (DSC, S-650 model, Sinco Co. Ltd., Seoul, Korea) and powder X-ray diffractometer (PXRD, D8 Advance X-ray diffraction system, Bruker AXS GmbH, Karlsruhe, Germany). DSC was calibrated for temperature and enthalpy using indium. The melting temperature and enthalpy of celecoxib and solid dispersion nanoparticles (2–5 mg) were then recorded at a heating rate of 5°/min under nitrogen purge (20 mL/min). Powder X-ray diffraction pattern was recorded from 5° to 50° of 2θ at a scanning rate of 3°/min. The celecoxib concentration within the solid dispersion nanoparticles was determined by dissolving about 30 mg of solid dispersion nanoparticles in 100 mL ethanol, filtering aliquots using a 0.45-μm syringe filter, and analyzing concentration with an HPLC system (Agilent 1100 Series, Agilent Technologies, Santa Clara, CA, USA). Chromatographic separation was performed with a CAPCELL PAK C18 UG120 (4.6 mm × 150 mm, 5 μm) reversed-phase column (Shiseido Fine Chemicals, Tokyo, Japan). Acetonitrile in water (60%) was used as the isocratic mobile phase at a flow rate of 1.0 mL/min. Celecoxib was detected by an ultraviolet (UV) detector at 238 nm. The drug content was calculated using the following equation: weight of the drug in nanoparticles/weight of the feeding excipients and drug × 100. Dissolution tests for raw celecoxib and celecoxib-PVP K30 solid dispersion nanoparticles were performed in 300 mL of a dissolution medium containing hydrochloric acid (HCl) and sodium chloride (NaCl) at pH 1.2 and 37 °C using a USP rotating paddle apparatus at 50 rpm (non-sink condition). Samples equivalent to 60 mg of celecoxib were added to the dissolution tester (Electrolab, Mumbai, India). At predetermined time intervals, 2 mL of the medium was sampled, filtered using a 0.45-μm glass fiber syringe filter, diluted with methanol, and analyzed for celecoxib concentration by HPLC as described above. Dissolution tests were also performed using acetate buffer (pH 4.0), phosphate buffer (pH 6.8) and water under the same conditions.

### 3.4. Solubility of Celecoxib in Surfactant Solution

To investigate the solubilization capability of gelucire 44/14, poloxamer 188, poloxamer 407, Ryoto sugar ester L1695, and TPGS, which were tested as surfactants, the solubility of celecoxib was measured in 1% surfactant solution at 37 °C. About 50 mg celecoxib was added to 5 mL of surfactant solution in glass vials. After sonication for 1 h, vials were placed in a shaking water bath at 100 rpm for 3 days. After setting solution aside for 4 h, 3 mL aliquots were filtered using a 0.45-μm syringe filter, diluted with methanol and then celecoxib concentration was analyzed by HPLC as described above.

### 3.5. Pharmacokinetic Study in Rats

The study protocol complied with the institutional guidelines for the care and use of laboratory animals, and it was approved by the ethics committee of Kyungsung University (No. 2014-04A). Oral bioavailability of celecoxib-PVP solid dispersion nanoparticles was evaluated in fasted Sprague-Dawley (SD) rats weighing 250 ± 10 g. Twenty SD rats were divided in to four groups (*n* = 5), and the first group was orally administered raw celecoxib, while the remaining three groups received solid dispersion nanoparticles of celecoxib-PVP K30, celecoxib-PVP K30-poloxamer 407, or celecoxib-PVP K30-TPGS, using an animal feeding needle at a dose of 100 mg/kg of celecoxib. Prior to oral dosing, the samples were dispersed in distilled water. Following a predetermined time interval, about 0.35 mL of blood was drawn from the retro-orbital plexus of the rats, collected in heparinized tubes, and centrifuged at 10,000 rpm for 5 min at 4 °C to obtain the plasma. The plasma concentration of celecoxibwas subsequently measured using liquid chromatography-tandem mass spectrometry (LC-MS/MS). Sample preparation and conditions for analysis adhered to previously reported methods [3,4,5]. For protein precipitation, 20 μL of a 20 μg/mL solution of hydrochlorothiazide (internal standard; IS) was added to 100 μL of heparinized plasma followed by 400 μL of acetonitrile. After vortexing briefly, the organic phase was separated from the aqueous phase by centrifugation at 13,000 rpm for 5 min. A 5-μL aliquot of the organic phase was injected into the LC-MS/MS system. Chromatographic separation was achieved using a ZORBAX^®^ Eclipse XDB-C 18 column (4.6 mm × 50 mm, 1.8 μm, Agilent Technologies). The HPLC system was operated isocratically at 40 °C. The mobile phase consisted of 0.1% formic acid:acetonitrile (25:75, *v*/*v*). The mass spectrometer (Agilent technologies 6410 triple quadrupole mass spectrometer) was equipped with an electrospray source. The ions monitored using multiple reaction monitoring were *m*/*z* 380 (parent) and *m*/*z* 316 (product) for celecoxib, and *m*/*z* 296 (parent), and *m*/*z* 205 (product) for the IS. Pharmacokinetic analysis of the data was carried out with WinNonlin Standard Edition software, Version 5.3 (Pharsight Corp., St. Louis, MO, USA). The area under the curve (AUC_0→24 h_) was calculated using the trapezoidal method. The maximum concentration of celecoxib after oral administration (C_max_) and the time to reach the maximum concentration (T_max_) were determined from the experimentally obtained data.

### 3.6. Statistical Analysis

The data were analyzed by a one-way analysis of variance (ANOVA) test followed by the Student-Newman-Keuls (SNK) and least-squares difference (LSD) tests using the SPSS 21.0 software (IBM SPSS Statistics, Armonk, NY, USA).

## 4. Conclusions

In this study, spherical celecoxib solid dispersion nanoparticles smaller than 300 nm were successfully developed using the SAS process. Among the formulations studied, celecoxib-PVP-TPGS solid dispersion nanoparticles showed significantly enhanced *in vitro* dissolution and oral absorption of celecoxib. In addition, *in vitro* dissolution efficiency was well correlated to *in vivo* pharmacokinetic parameters (C_max_, and AUC). The present study therefore demonstrated that the formulation of celecoxib-PVP-TPGS solid dispersion nanoparticles using the SAS process is a highly effective strategy for enhancing the bioavailability of poorly water-soluble celecoxib.
